# Genetic Diversity of Kazakhstani *Equus caballus* (Linnaeus, 1758) Horse Breeds Inferred from Microsatellite Markers

**DOI:** 10.3390/vetsci10100598

**Published:** 2023-09-30

**Authors:** Zarina Orazymbetova, Daniya Ualiyeva, Kairat Dossybayev, Aibyn Torekhanov, Dauren Sydykov, Aizhan Mussayeva, Gabiden Baktybayev

**Affiliations:** 1 Kazakh Research Institute of Livestock and Fodder Production, Almaty 050035, Kazakhstan; orazymbetova.z@gmail.com (Z.O.); day7861@mail.ru (D.S.);; 2 Institute of Genetics and Physiology, Committee of Science of the Ministry of Science and Higher Education of the Republic of Kazakhstan, Almaty 050060, Kazakhstan; 3 Institute of Zoology, Committee of Science of the Ministry of Science and Higher Education of the Republic of Kazakhstan, Almaty 050060, Kazakhstan; 4 Chengdu Institute of Biology, Chinese Academy of Sciences, Chengdu 610041, China

**Keywords:** domesticated horse, STR loci, genetic differentiation, population structure

## Abstract

**Simple Summary:**

Traditional horse breeding has long developed several kinds and lineages of horse breeds in Kazakhstan. Among them, Kushum and Mugalzhar are the breeds most prolific and resistant to harsh climatic conditions. Microsatellite analysis is employed to examine present genetic variability and population structure. The subpopulation structure shows three regional groups indicating (I) purebred Kushum populations from western Kazakhstan, (II) the Kozhamberdy type of Mugalzhar interbreed in populations from central Kazakhstan, and (III) admixed Kushum–Mugalzhar populations from western and southwestern Kazakhstan. The majority of microsatellite markers utilized are informative, with seven of them being extremely variable. A high level of genetic admixture among Kazakhstani *Equus caballus* breeds is found, as well as a shallow level of genetic differentiation between the examined populations.

**Abstract:**

Understanding the genetic diversity and structure of domesticated horse (*Equus caballus*) populations is critical for long-term herd management and breeding programs. This study examines 435 horses from Kazakhstan, covering seven groups in three geographic areas using 11 STR markers. Identified are 136 alleles, with the mean number of alleles per locus ranging from 9 to 19. VHL20 is the most variable locus across groups, while loci HTG4, AHT4, AHT5, HTG7, and HMS3 are variable in most populations. The locus AHT5 in the Emba population shows the highest frequency of rare alleles, while the lowest frequency, 0.005, is observed in the Kulandy population. All loci were highly informative for the Kazakhstani populations of *E. caballus*, with PIC values higher than 0.5. Pairwise variations in Wright’s F_ST_ distances show that the examined varieties have little genetic differentiation (0.05%), indicating a high degree of admixture and a continuing lineage sorting process. Phylogenetic and population structure analyses reveal three major clusters of Kazakh horses, representing (I) the Uralsk population of the Kushum breed and the monophyly of two groups: (II) the Kozhamberdy population of the Mugalzhar breed, and (III) the Mugalzhar–Kushum breed populations. Kazakhstani horse populations, while being regionally isolated, were recently in contact with each other.

## 1. Introduction

The horse was the last domesticated animal which had a long-term significant and enduring impact on human civilizations, leading to advancements in transportation and trade, influencing people’s lifestyle, and revolutionizing the nature of warfare. In turn, human selection changed genetic diversity in horse populations, resulting in the variation seen among current horse characteristics and types. Domestic horses were subject to constant genetic restocking from the wild, mostly from females [1,2,3,4].

Kazakhstan, the largest Central Asian country, combines several climatic and geomorphologic features that offer an optimal environment for agricultural growth due to its geographical location at the intersection of the western and eastern Palearctic. The FAO database (Domestic Animal Diversity Information System, accessed on 5 December 2022), identifies Kazakhstan as having eight registered domesticated horse breeds (Kushum, Mugalzhar, Kostanay, Akhal-Teke, Aday, Kazakh, Jabe, and Karabair) [5]. Among them the Kushum breed was formed via purebred crossbreeding, whereas the Mugalzhar breed was established by traditional crossing methods, and these two breeds are dedicated to meat and milk production. The dairy production presented by fermented mare’s milk called “Kymyz” and fresh milk “Saumal” are national drink products of Kazakhstan. They are rich in minerals and vitamins, and benefit human health with anticancer and anti-inflammation effects [6]. The overall number of horses in Kazakhstan now surpasses 3 million, with 90% of them raised by herds in practically all areas of the country [7].

The Kushum horse was bred by the herd method of Ural and Aktobe farms, named after the Kushum River, flowing across western Kazakhstan. Initial approval as a separate breed came in 1976. The breed was established by a complex reproductive crossover procedure. Trotting, Thoroughbred riding, and Don breeds were crossed with local native mares [8]. The farmers chose the best animals in terms of growth and maintained them on semi-desert cereal–wormwood pastures throughout the year. As a result, Kushum horses are ideally suited for herding. Crosses and rigorous selection of stallions for desired traits were carried out in the middle of the twentieth century in terms of external features and adaptation to local environments. Later, the crossed horses were bred “in themselves” to reinforce breeding results [9].

Mugalzhar horses are an upgraded dual-purpose form of the Kazakh horse breed Jabe type, developed by Kazakhstani scientists between 1969 and 1998 [10]. They are distinguished by high-quality meat and milk production, as well as resilience to extreme weather conditions and year-round grassland farming [11].

Many studies have been conducted on a global scale in the horse breeding sector involving research of mtDNA analysis of modern horses [12,13,14,15,16] and fossils [17,18]. Microsatellite analysis has been widely utilized for parentage testing [19,20,21], as well as a single nucleotide polymorphism (SNP) analysis to determine the population structure and genetic variability of Equids [22,23,24].

Genetic distinctiveness can be clarified by population genetic structure, leading to breed preservation, including future breeding methods and management plans. Microsatellites have been a widely utilized genetic marker and effectively applied to studies of inter- and intrabreed variability in domestic and feral horse populations [25,26,27,28,29,30,31,32].

Here we determine the genetic diversity of the Kushum and Mugalzhar horse breeds within populations and between them. Applied are several different approaches to evaluate the distribution of molecular indices, genetic distances, and population structural variation, which can elucidate lineage sorting processes, and be helpful for organizing individual genetic feature panels of the type or line as well as to improve the inbreeding management strategy of Kazakh horse-breed populations.

## 2. Materials and Methods

### 2.1. Sampling

**Figure 1:**
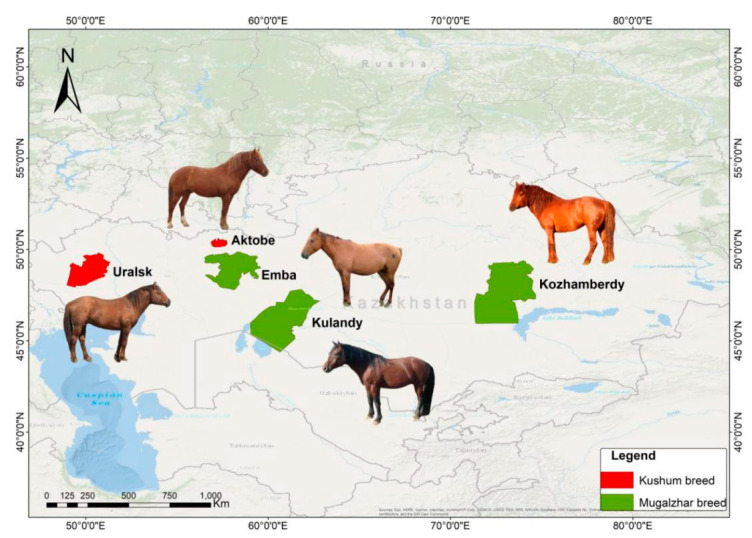
Distribution map of two main Kazakh horse breeds, Kushum and Mugalzhar.

DNA isolation of 435 samples from blood cohorts were taken from 7 populations across Kazakhstan, representing Mugalzhar and Kushum horse breeds (Table 1; Figure 1).

### 2.2. Laboratory Protocol

A panel of 11 highly polymorphic microsatellite loci (AHT4, AHT5, ASB2, ASB23, HMS3, HMS6, HMS7, HTG4, HTG7, LEX3, and VHL20) were chosen for typing using methods described by Zabek and Fornal (2009) [33].

DNA extractions were conducted using the “DNA-sorb-B” set (AmpliSens, Moscow, Russia). Biomaterial was processed by the solid-phase sorption method, which consists in adding a lysing solution, DNA sorption on a sorbent, repeated washing and resorption of DNA with a buffer solution, as a result of which a purified solution containing DNA was obtained. Furthermore, the spectrometric quantification was performed as well as amplification reactions carried out using the StockMarks Equine Kit (Applied Biosystems, Waltham, MA, USA) [34]. Separation and analysis of amplified fragments were carried out by capillary electrophoresis using a genetic analyzer. PCR amplifications were performed on Thermocycler 2730 (Applied Biosystems) following a touchdown cycling protocol with an initial denaturation at 95 °C for 15 min, followed by 30 cycles of: the first 4 cycles, 58 °C (30 s), 59 °C (120 s), 72 °C (75 s); the next 6 cycles, 94 °C (30 s), 59 °C (120 s), 72 °C (75 s); the next 20 cycles, 90 °C (30 s), 59 °C (120 s), 72 °C (75 s); ending in a 5 min extension at 68 °C, with a 4 °C hold temperature. Amplification product separation was performed by capillary electrophoresis on an automatic genetic analyzer AB 3130 (Applied Biosystems), using the GeneMapper™ v. 4.0 program. Amplified DNA fragments were interpreted using a control DNA profile with a known genotype and data from international comparative tests (Horse Comparison Tests) conducted by ISAG.

### 2.3. Population Genetic Structure

Allele frequencies and polymorphic information content (PIC) were calculated using Cervus 3.0 software [35,36]. Genetic diversity within and between breeds, as well as basic parameters, including total number of allele variants (NA), effective number of alleles (NE), estimation of observed (HO), expected (HE), and unbiased expected (UHE) heterozygosity, and Shannon’s information index (I) were measured using GenAlEx 6.5 software (New Brunswick, NJ, USA) [37]. Variance components of microsatellite diversity within and between populations for all pairs of populations were analyzed using analysis of molecular variance (AMOVA) with permutations set to 999 in the GenAlEx 6.5 [37]. Chi-square tests of Hardy–Weinberg equilibrium and rare alleles were calculated for each population using Microsatellite Analyzer v. 4.05 (MSA) [38]. Fixation indices (F_IT_, F_IS_, and F_ST_) of Wright’s F-statistics were obtained using GenAlex 6.5 and Excel microsatellite toolkit (version 3.1) [37]. Neighbor joining of Saitou and Nei (1987) [39] was used to construct a phylogenetic tree based on Nei’s genetic distance in MEGA 7 [40]. Factorial correspondence analysis (FCA) was investigated based on the individual multilocus genotype using GENETIX version 4.03 [41]. Bayesian clustering analysis was implemented in Structure 2.3.4 [42] without prior structure information. All possibilities were considered by dividing 7 populations into 7 groups. An ad hoc quantity based on the second order rate of change in the likelihood function with respect to K (K) was used for estimating the number of clusters from structure analysis [43]. In addition, we also use ln(Pr(X|K) values in order to identify the k for which Pr(K = k) is highest (as described in STRUCTURE’s manual, Section 5.1. Twenty runs for K = 1 to 7 were analyzed under the admixture model, correlated allele frequencies, and a burn-in of 250,000 followed by 1,000,000 Markov chain Monte Carlo (MCMC) iterations. Structure Harvester 0.6.93 [44] was applied to choose the optimal K-value based on the Delta K method. The 20 replicates for the chosen K-value were merged using CLUMPP 1.1.2 [45] and the final plots were generated using DISTRUCT 1.1 [46].

## 3. Results

### Microsatellite Genotyping, and Population Genetic Diversity and Structure

A total of 136 alleles at 11 STR loci from 435 genotyped individuals of two Kazakh horse breeds from seven populations were identified. All markers were found to be polymorphic (*p* ≥ 0.05) (Table 2). The mean number of alleles varied from 9 at loci AHT4, HMS6, and HMS7 to 19 at locus ASB23. The mean number of alleles (Na) per locus was 12.36, and the effective number of alleles (Ne) was 5.82. The expected heterozygosity (He), which is a widely accepted measure of genetic diversity in a population, ranged from 0.64 in locus HTG4 to 0.83 in locus VHL20, with an average He of 0.77 across the seven populations for the 11 microsatellite loci analyzed. The observed heterozygosity (Ho) fluctuated from 0.48 in locus LEX3 to 0.85 in locus VHL20, with a population mean of 0.68, indicating that all studied lineages are characterized by considerable genetic variability. The polymorphic information content (PIC) varied from 0.62 for the marker ASB2 to 0.82 for the AHT4 locus. The average PIC for the 11 microsatellite markers was 0.74 and there were no markers with a PIC of less than 0.5, indicating that all loci were found to be highly polymorphic. Shannon’s information (diversity) index (I), which is an indicator of the genetic variability of a population, ranged from 1.33 in locus HTG4 to 1.99 in the VHL20 marker. The average value of the I-index for all seven populations was equal to 1.73, which reflects the level of allele abundancy (Table 2). Further, F_IS_, F_IT_, and F_ST_ indices were calculated for each marker in whole populations. F_IS_ ranged from −0.031 (VHL20) to 0.093 (AHT4) with an average value of 0.211 for all loci. F_IT_ presented a mean value of 0.157 ranging from −0.010 for HTG7 to 0.439 for LEX3. The calculation of FIS was between 0.030 (AHT5) and 0.074 (ASB2) with a mean value of 0.041 in the total population.

AMOVA analysis performed on seven populations, suggests that the majority of the variation occurred within individuals—70% (Table 3). Fixation indices based on standard permutation demonstrated differences (*p* ≥ 0.001) indicating a reduction of heterozygosity, panmixia, and inbreeding processes which occurred in Kazakhstani populations of *E. caballus*.

Across Kazakhstan, horse breeds are identified with rare alleles that are typical for each population: VHL20, HTG4, HMS3, HMS6, HMS7, AHT4, AHT5, ASB2, ASB23, and LEX3. Among them, five unique alleles were observed in the Uralsk (Population 1) and Kulandy (Population 7). For the Uralsk population unique alleles were found in the HTG locus at 121 bp and 125 bp lengths, at HMS6 at 155 bp length, and HMS7 169 bp length. For the Kulandy population a unique allele was found in the locus LEX3 143 bp length. 

The value for gene differentiation based on F-statistic (F_ST_) distance over all loci between populations of the Kushum breed (Uralsk with Aktobe) was 4.5%, whereas between populations of the Mugalzhar breed it varied in the range of 0.008–2.8%. Genetic variability between the Uralsk population and Mugalzhar populations was from 3.3% to 4.7%, which indicates that 4.7% of the variability could be attributed to differences between breeds (Table 4). A chi-squared test observed statistically significant (*p* ≤ 0.05) results at all loci, rejecting the null hypothesis of random mating (Table S1).

**Figure 2:**
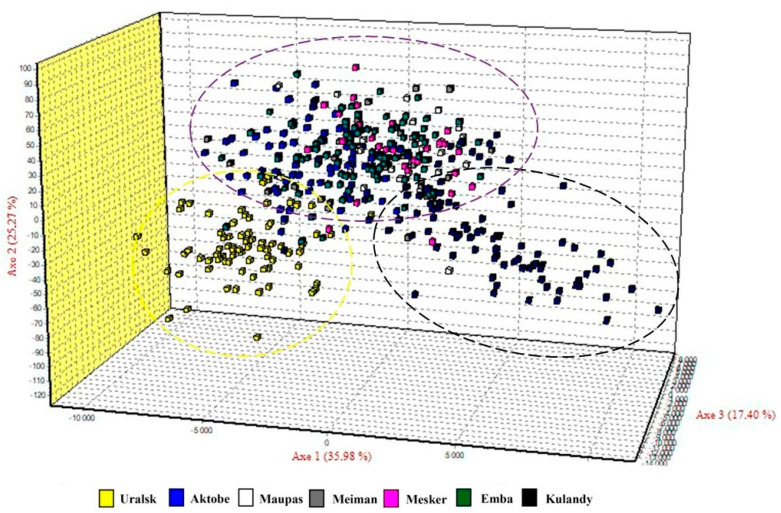
Factorial correspondence analysis of 7 horse populations studied on the basis of 11 STR loci. Dashed lines representing the three clusters with the following colors: yellow—Uralsk, Kulandy—black, and waterloo color—Aktobe, Emba, Maupas, Meiman, Mesker.

Factorial correspondence analysis revealed three clusters of horse populations are distinct at three axes with variance of 35.98%, 25.27%, and 17.40%, respectively (Figure 2). An FCA plot demonstrated that the Uralsk population of the Kushum breed was clearly separated from other horses and thus the result is consistent with the phylogenetic tree and structure inferences.

**Figure 3:**
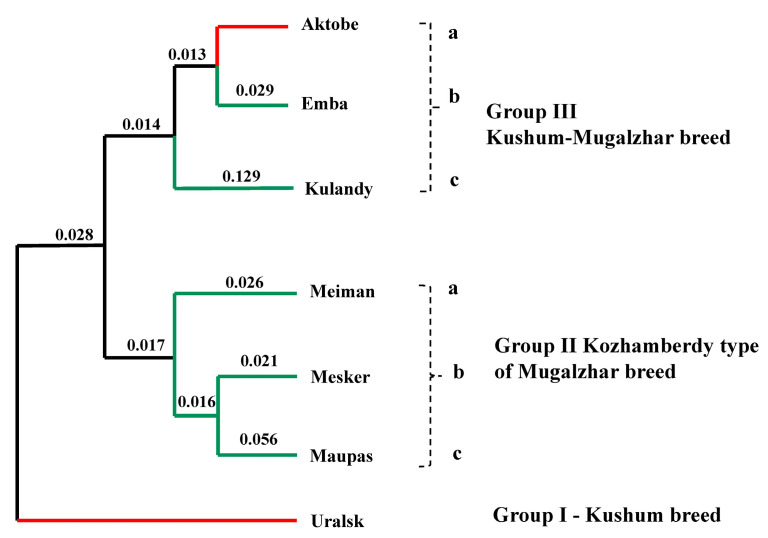
Neighbor-joining dendrogram showing the relationships of seven Kazakh horse populations with Nei’s genetic distances plotted. Outline colors of the tree branches representing the breeds according to Figure 1; red = Kushum breed, green = Mugalzhar breed.

An unrooted neighbor-joining tree for all samples was constructed using a pairwise population matrix of Nei’s genetic distances in order to represent relationships among seven populations of Kazakh horse breeds (Figure 3). Three main groups were recovered: Group I is the Uralsk population with a distance of 2.8% to monophyletic groups II and III. Group II is the Kozhamberdy type population representing three lines: IIa, Meiman; IIb, Maupas; and IIc, Mesker (1.4%). Group III clusters members of two breeds consisting of IIIa, Aktobe (Kushum breed); IIIb, Emba (Mugalzhar breed); and IIIc, Kulandy type populations (Mugalzhar breed) (1.6–1.7%).

**Figure 4:**
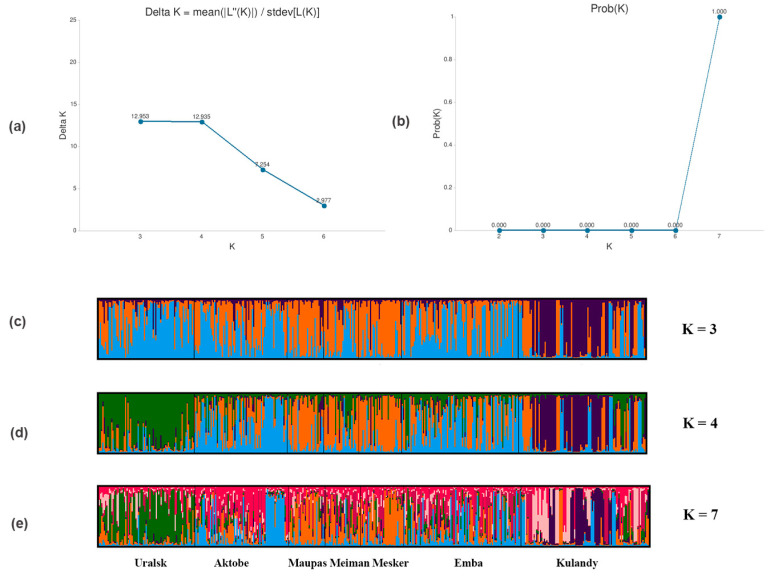
The best K results of the hierarchical STRUCTURE clustering. (**a**) Method of [33]; (**b**) method of [32]; (**c**) variance of K = 3; (**d**) K = 4; (**e**) K = 7. The green color representing the Uralsk population, blue color − Aktobe and Emba populations, orange color − Kozhamberdy type (Maupas, Meiman, Mesker) populations, blue color − Kulandy population.

Bayesian cluster analysis performed with STRUCTURE [33] showed that the independent runs from K = 2 to K = 7 produced consistent results, where the most likely K values were identified at K = 3 and 4 (ΔK = 12.953; 12.935), respectively (Figure 4); the subpopulations’ structure [32] using the median values of Ln Prob of data to calculate Prob(K = k) yielded the uppermost value of K = 7. A plot with the clustering of individuals is presented in Figure S1.

## 4. Discussion

A comprehensive genetic analysis of microsatellite markers conducted for seven populations of two main horse breeds of Kazakhstan revealed a high genetic diversity. Wright’s F_ST_ analysis revealed a genetic differentiation of 4.5% between subpopulations of the Kushum breed, while to the Kulandy population of Mugalzhar breed the genetic distance was 4.7%. Nonetheless, the level of genetic variability between the Uralsk and Aktobe populations was determined to be weak, with *p* ≤ 0.05. This might be explained by the attribution to the same breed and possible stallion interchange given their geographic proximity.

The mean number of alleles in this study was 9 at locus AHT4 which is slightly lower than in Danubian horses (11.2) [47]. The same locus was characterized by high PIC values (0.82), which is in concordance with Halla horses [48]. The average of expected heterozygosity across the loci was computed at 0.77 in Kazakh horses and Mugalzhar horses [27], and 0.8 in Mongolian horses, which was higher than in Thoroughbred horses (0.72) [48]. The minimum observed heterozygosity was identified at locus LEX3 (0.479), where notably the same finding was reported in the study of [27].

The subpopulation inbreeding coefficient relative to the whole population showed that genetic differentiation is higher between breeds, compared to subpopulations [49]. Comparable results were found in Algerian horse breeds, which are not isolated breeds [50]. In the present study, F_ST_ findings were considerably higher than those in Bhutan horse breeds (0.003–0.008) [51], Turkish Rahvan (0.0019) [52], and Guizhou and Luoping (0.001) horse breeds [53]. Phylogenetic relationships recovered three main groupings representing (I) the Uralsk population, (II) the Mugalzhar populations, and (III) admixed populations of Kushum and Mugalzhar. Purebred Mugalzhar populations (II) of Meiman (IIa), Maupas (IIb), and Mesker (IIc) have a demonstrated relatedness of 1.6–1.7%. In contrast, a monophyletic group of admixed (IIIa) Kushum and (IIIb, c) Mugalzhar breed populations has a genetic distance of 2.8%. The Emba (IIIb) and Kulandy (IIIc) populations together group with the Aktobe (IIIa) population of the Kushum breed and have a difference in similarity of 1.3–1.4%.

Cluster analysis results [42] reveal the substructure of populations based on Δ K variation, which suggest the existence of three groups, also supported by phylogenetic analysis and FCA as described in Section 3. However, another approach of using the median values of Ln Prob is to calculate Prob(K = k) [43] detected for the possible number of subpopulations equal to seven. This may be explained when alpha is close to zero, which means most individuals are essentially from one population or another; however, when alpha ≥ 1, it means that most individuals are admixed [54]. On the other hand, STRUCTURE analysis indicates differences within subpopulations of studied horses. At K = 4 and K = 7 it clearly represents the difference between Uralsk and Aktobe populations despite being from the same breed. This is not a real surprise. It might be the result of the short history of the breed and the different initial mare populations. These findings are consistent with the results of phylogenetic analysis.

Further, Kulandy-type horses (IIIc) revealed a different genetic background in all K, while almost the same ancestral component was distinguished in the three lines of Kozhamberdy horses (II). At all values of K, a more similar pattern was observed between Aktobe (IIIa) and Emba (IIIb) populations. In the present study, the horse breeds, types and lines are also phenotypically diverse, sampled from different locations and various pasture areas. For instance, depending on the natural, climatic, and forage conditions of breeding, the Mugalzhar breed is divided into three types: Kozhamberdy, Emba, and Kulandy. Kozhamberdy forms the type of horses established in the Karagandy region of central Kazakhstan, with Emba from the Aktobe region of western Kazakhstan, and the Kulandy type of horses produced in the Kyzylorda region of the southwest part of the country. Moreover, this type of horse has high meat and milk productivity, and is well adapted to harsh environmental conditions, showing characteristic features of main breeding traits and steadfastly passing them on to their offspring.

Taking into account that the biological unit in domesticated animals is usually breed, differences of genetic traits among individuals characterize the endurance and adaptability of an organism. The STR loci are a universal tool for the examination of genetic variability among horse breeds [33,55,56]. Further, to examine genetic variability within and between breeds, the observed and expected heterozygosity were estimated. Results indicate a higher degree of related mating of individuals in a subpopulation and are inconsistent with previous studies [27]. To the best of our knowledge, herd farmers, to improve herds, usually use the most productive stallions on a farm without control of their origin. This may cause a low level of genetic variation in studied populations.

In the present study among identified rare alleles, private alleles were detected in loci HTG4, HMS6, and HMS7 for the Uralsk (Population 1), and in the locus LEX3 for the Kulandy (Population 7), which is highlighting their favor in genetic differentiation from each other [57,58,59]. Highly variable alleles for all populations were found in the locus VHL20, as well as the HTG4 locus for Populations 1–5 and 7; AHT4 for Populations 3 and 7; AHT5 for Populations 1–2 and 5–7; HTG7 for Populations 1–3 and 5–7; and locus HMS3 for Populations 3–4. This is consistent with Zaitcev et al. (2021) [59], where loci VHL20 and HTG4 were highly variable for the Priobskaya Russian horse breed population. The mean of observed heterozygosity was (0.68) similar to identified values of Lipizzaner (0.68) and Thoroughbred (0.68) horses. However, the mean of expected heterozygosity (0.77) was higher than those of Lipizzaner (0.69) and Thoroughbred (0.69) [60,61,62]. As a result, the calculated observed and expected heterozygosity showed that the studied populations are under inbreeding. Moreover, these results were confirmed by the F-statistics findings that F_IT_, F_IS_, and F_ST_ reflected a reduction of heterozygosity and weak divergency (F_ST_ ≤ 0.05) in the populations [63]. Confirmation is acquired here that the STR loci are appropriate for assessing genetic diversity and variation within and among Central Asian horse populations.

Considering the fact that the SNP (single nucleotide polymorphisms) genotyping approach has been widely applied to the investigations of Equines’ population structure [64,65], yet has a low mutation rate and abundance in genome compared to the microsatellite markers, we recommend continuing the study in the perspective of genome-wide SNP analysis to fulfill the whole picture of the population structure and genetic diversity of the Kazakh horses.

## 5. Conclusions

In the present study, microsatellite analysis allowed us to identify a division of the Uralsk population from populations of Aktobe (Kushum breed) and Kulandy (Mugalzhar breed), the latter of each differentiated by 4.5% and 4.7%, respectively. Moreover, a significant degree of population admixture between breeds and within types and lines of Kazakhstani *Equus caballus* was detected. STR loci were shown to be particularly helpful for characterizing the allele pool and assessing the extent of genetic differentiation of studied populations. Assessment of genetic variation and F-statistics presented a reduction of heterozygosity in all populations. Conducting a comparative microsatellite typing is critical for monitoring genetic variability and tracing patterns in the selection process. Furthermore, data on genetic diversity computed within and across breed variants might give insight into future conservation initiatives and better horse breeding management.

## Figures and Tables

**Table 1:** Sampling information of studied populations of *E. caballus*.

N	Population	Ns	Location
Kushum breed			
1	Uralsk type	76	Zhangali district, W KZ
2	Aktobe type	74	Aktobe region, W KZ
Mugalzhar breed			
6	Emba type	93	Aktobe region, W KZ
7	Kulandy type	101	Kyzylorda region, SW KZ
Kozhamberdy type			
3	Maupas interbreed line	29	Karaganda region, C KZ
4	Meiman interbreed line	28	Karaganda region, C KZ
5	Mesker interbreed line	34	Karaganda region, C KZ

N—consecutive number of populations; Ns—number of samples; W—western; SW—southwestern; C—central; KZ—Kazakhstan.

**Table 2:** Summary statistics of mean genetic diversity at 11 microsatellite loci in 435 individuals of *E. caballus* from Kazakhstan.

Locus	N_A_	N_E_	I	H_O_	H_E_	UH_E_	F_IS_	F_IT_	F_ST_	PIC
VHL20	10.57	6.026	1.991	0.855	0.830	0.839	−0.031	0.004	0.034	0.813
HTG4	6.423	2.900	1.338	0.634	0.642	0.650	0.013	0.044	0.032	0.629
AHT4	8.429	5.701	1.867	0.746	0.823	0.832	0.093	0.125	0.035	0.822
HMS7	6.571	3.911	1.496	0.667	0.728	0.736	0.083	0.112	0.031	0.734
AHT5	7.000	4.908	1.695	0.779	0.796	0.805	0.021	0.051	0.030	0.784
HMS6	7.571	4.660	1.717	0.652	0.783	0.793	0.168	0.206	0.046	0.788
ASB23	9.857	5.678	1.918	0.502	0.822	0.837	0.389	0.415	0.042	0.713
ASB2	10.00	6.334	1.927	0.842	0.806	0.896	−0.045	0.033	0.074	0.627
HTG7	7.000	3.568	1.453	0.751	0.712	0.720	−0.054	−0.010	0.041	0.686
HMS3	8.143	4.676	1.711	0.566	0.784	0.793	0.278	0.306	0.040	0.786
LEX3	9.571	5.728	1.912	0.479	0.814	0.825	0.422	0.439	0.047	0.768
Mean	8.286	4.917	1.730	0.679	0.776	0.793	0.121	0.157	0.041	0.741

Numbers of observed alleles (N_A_), number of effective alleles (N_E_), Shannon’s information index (I), observed heterozygosity (H_O_), expected heterozygosity (H_E_), unbiased expected heterozygosity (UH_E_), inbreeding coefficient (F_IS_), fixation index (F_IT_), population differentiation statistic (F_ST_), *p* ≤ 0.001.

**Table 3:** Analysis of molecular variance.

Source of Variation	d.f.	SS	MS	Est. Var.	% of Variation
Among populations	6	240.411	40.069	0.290	6%
Among individuals	428	2270.183	5.304	1.074	24%
Within individuals	435	1372.500	3.155	3.155	70%
Total	869	3883.094		4.520	100%
F_ST_ = 0.064 (*p* ≥ 0.001); F_IS_ = 0.254 (*p* ≥ 0.001); F_IT_ = 0.302 (*p* ≥ 0.001)					

d.f.—degrees of freedom; SS—sum of squares; MS—mean of squares; Est. Var.—estimated variance.

**Table 4:** Genetic distances of studied horse populations.

	Uralsk	Aktobe	Maupas	Meiman	Mesker	Emba	Kulandy
Uralsk							
Aktobe	0.045						
Maupas	0.037	0.021					
Meiman	0.035	0.020	0.008				
Mesker	0.041	0.021	0.010	0.014			
Emba	0.033	0.014	0.014	0.014	0.021		
Kulandy	0.047	0.025	0.022	0.021	0.028	0.021	

The Wright’s F_ST_ fixation indices given below diagonal.

## Data Availability

Not applicable.

## Supplementary Materials

The following supporting information can be downloaded at: https://www.mdpi.com/article/10.3390/vetsci10100598/s1, Figure S1: Estimation of the population structure with different K values (K = 2 to K = 7). Table S1: Chi-squared test of Hardy–Weinberg equilibrium for the 11 STR loci.
