# Supplementary material for: Genetic Diversity of Kazakhstani Equus caballus (Linnaeus, 1758) Horse Breeds Inferred from Microsatellite Markers
Source: Vet Sci. 2023 Sep 30;10(10):598. doi: 10.3390/vetsci10100598 (PMC10611244; doi:10.3390/vetsci10100598)
Supplement: Supplementary file 1 [file vetsci-10-00598-s001.zip › vetsci-2603439-supplementary.pdf]

Supplementary Materials

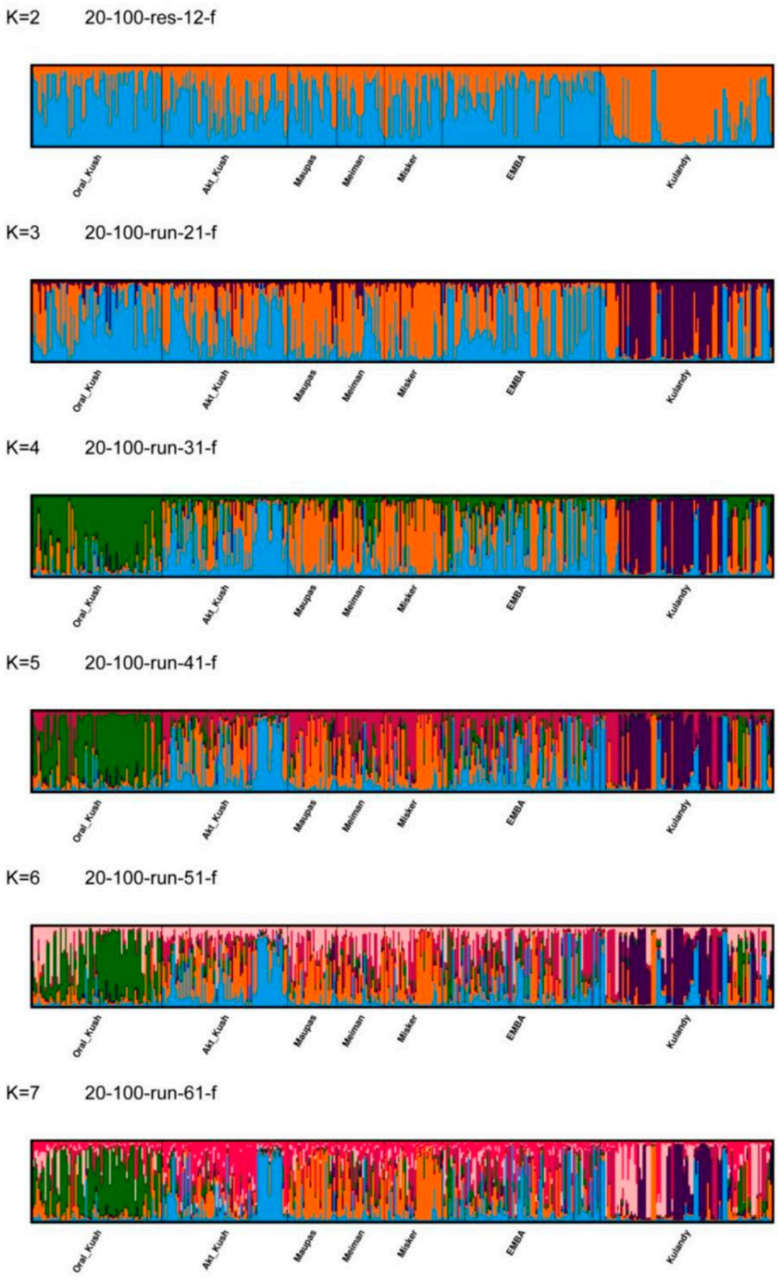

Figure S1. Estimation of the population structure of Kazakhstani horse breeds with different K values (K=2 to K=7).

Table S1. Chi-squared test of Hardy-Weinberg equilibrium for the 11 STR loci.

Locus	d.f.	Chi-sq.	Prob.	Signif.
VHL20	91	155.836	0.000	***
HTG4	36	486.012	0.000	***
AHT4	66	168.521	0.000	***
HMS7	36	79.986	0.000	***
AHT5	66	473.456	0.000	***
HMS6	36	509.129	0.000	***
ASB23	171	1742.997	0.000	***
ASB2	153	408.975	0.000	***
HTG7	45	125.891	0.000	***
HMS3	66	593.617	0.000	***
LEX3	66	1085.16	0.000	***

D.f. - degree of freedom; Chi-squared test value; probability; significance - *** (if $p \leq 0.05$).
